# Hearing rehabilitation of adults with auditory processing disorder: a systematic review and meta-analysis of current evidence-based interventions

**DOI:** 10.3389/fnhum.2024.1406916

**Published:** 2024-06-21

**Authors:** Rachel Crum, Sanathorn Chowsilpa, Diego Kaski, Paola Giunti, Doris-Eva Bamiou, Nehzat Koohi

**Affiliations:** ^1^The Ear Institute, University College London, London, United Kingdom; ^2^Otology Neurotology and Communication Disorder Unit, Department of Otolaryngology, Faculty of Medicine, Chiang Mai University, Chiang Mai, Thailand; ^3^Department of Clinical and Movement Neurosciences, Institute of Neurology, University College London, London, United Kingdom; ^4^Neuro-otology Department, University College London Hospitals, London, United Kingdom; ^5^Ataxia Centre, National Hospital for Neurology and Neurosurgery, University College London Hospitals, London, United Kingdom; ^6^Biomedical Research Centre, National Institute for Health Research, London, United Kingdom

**Keywords:** auditory processing disorder, auditory training, low-gain hearing aids, personal remote microphone system, speech in noise perception

## Abstract

**Background:**

For adults with auditory processing disorder (APD), listening and communicating can be difficult, potentially leading to social isolation, depression, employment difficulties and certainly reducing the quality of life. Despite existing practice guidelines suggesting treatments, the efficacy of these interventions remains uncertain due to a lack of comprehensive reviews. This systematic review and meta-analysis aim to establish current evidence on the effectiveness of interventions for APD in adults, addressing the urgent need for clarity in the field.

**Methods:**

Following the Preferred Reporting Items for Systematic reviews and Meta-Analyses (PRISMA) guidelines, we conducted a systematic search across MEDLINE (Ovid), Embase (Ovid), Web of Science and Scopus, focusing on intervention studies involving adults with APD. Studies that met the inclusion criteria were grouped according to intervention with a meta-analysis only conducted where intervention, study design and outcome measure were comparable.

**Results:**

Out of 1,618 screened records, 13 studies were included, covering auditory training (AT), low-gain hearing aids (LGHA), and personal remote microphone systems (PRMS). Our analysis revealed: AT, Mixed results with some improvements in speech intelligibility and listening ability, indicating potential benefits but highlighting the need for standardized protocols; LGHA, The included studies demonstrated significant improvements in monaural low redundancy speech testing (*p* < 0.05), suggesting LGHA could enhance speech perception in noisy environments. However, limitations include small sample sizes and potential biases in study design. PRMS, Demonstrated the most consistent evidence of benefit, significantly improving speech testing results, with no additional benefit from combining PRMS with other interventions.

**Discussion:**

PRMS presents the most evidence-supported intervention for adults with APD, although further high-quality research is crucial for all intervention types. The establishment and implementation of standardized intervention protocols alongside rigorously validated outcome measures will enable a more evidence-based approach to managing APD in adults.

## 1 Introduction

### 1.1 Auditory processing disorder—definition

Auditory processing disorder (APD), also known as central auditory processing disorder (CAPD), stems from neural dysfunction in the central auditory nervous system (CANS) and involves difficulties in interpreting speech and non-speech signals (British Society of Audiology, 2018). The CANS is a network of neural fibers spanning from the cochlear nucleus at the pontomedullary junction in the brainstem to the auditory cortex on the temporal lobe and extending to secondary auditory cortices (Bamiou et al., 2001). Dysfunction of the CANS network may occur due to a lack of synchrony of the firing neurons, decreased central inhibition or lesions at any point along the pathway (Bamiou et al., 2001). Any disruption in auditory processing (AP) will result in hearing deficits, especially in an environment of competing noise.

According to the (British Society of Audiology, 2018), APD is categorized into three groups: (1) Developmental APD—cases arise in children with normal audiograms and no known etiology, (2) Acquired APD—the listening difficulties are linked to an event e.g., aging process or a stroke, and (3) Secondary APD—the cases are linked to a temporary or permanent peripheral hearing loss e.g., glue ear.

### 1.2 Diagnosing APD

Diagnosis of APD is complex and a multidisciplinary team is required to accurately identify the presence of auditory processing disorder. The assessment may include, in addition to a thorough history, a range of specialist AP behavioral tests, full audiometric testing, immittance testing, speech testing in quiet and in background noise, otoacoustic emissions (OAEs), neurological auditory brainstem response (ABR), a speech and language assessment and/or cognitive or other assessments depending on the patient presentation (Chermak et al., 2017). Symptoms *per se* are not diagnostic for APD (Iliadou et al., 2017). There is no worldwide accepted “gold standard” testing battery, APD encompasses a range of clinical presentations and presumably different pathophysiological mechanisms (British Society of Audiology, 2018). However, using APD as an umbrella term for patients with “listening difficulties” or “suspected APD” without documenting the presence of deficits in appropriately validated tests renders intervention studies incomparable, as the participants reported symptoms may primarily be due to non-auditory disorders. Therefore, key diagnostic criteria have been suggested (ASHA, 2005a; American Academy of Audiology, 2010; Iliadou et al., 2017) and include: results at or below two standard deviations (SD) below the mean in at least two validated AP tests, a hearing threshold of ≤ 15 dB in both ears across the frequency range 250–8,000 Hz, and have a non-verbal IQ of >80. These have yet to be adopted worldwide.

### 1.3 Presentation and etiology in adults

Patients often (but not always) have normal pure tone thresholds without middle ear pathology and normal outer hair cell function but struggle with functional hearing particularly in the presence of background noise. Adults may also have difficulty in following multiple auditory instructions, distinguishing sounds, localizing, tracking, and grouping sounds and frequently mishear words (Iliadou et al., 2017). A decreased ability to appreciate music and difficulties learning new languages or technical jargon has also been observed (Chermak et al., 2017). A history of childhood academic struggles may indicate undiagnosed developmental APD (Baran, 2014).

Neurological disorders are a known cause of APD in adults. Epilepsy (Han et al., 2011), dementia (Sardone et al., 2020), cerebrovascular disease [e.g., stroke (Koohi et al., 2017a)], migraine (Agessi et al., 2014), Friedreich's ataxia (Teive et al., 2021), multiple sclerosis (Valadbeigi et al., 2014), Parkinson's disease (Guehl et al., 2008), neuro-infections, brain tumors, traumatic brain injury (TBI) (Bergemalm and Lyxell, 2005; Gallun et al., 2012), and metabolic disorders (Kaga et al., 1980) are all known to disrupt the neural networks extending into the CANS. Patients with evidence of brain pathology also have poor performance on temporal ordering and temporal resolution tests (Chowsilpa et al., 2021). Aging has been shown to particularly affect temporal processing abilities with some suggesting this occurs as early as middle age (Kumar and Sangamanatha, 2011; Sardone et al., 2020). Furthermore, central auditory dysfunction has been reported in neurodegenerative dementias such as Alzheimer's disease, Lewy body disease and frontotemporal dementia and that the degeneration of central auditory processing mechanisms will likely amplify any degree of peripheral hearing impairment and reduce the listening ability in noisy conditions (Johnson et al., 2021). Auditory processing disorder is also reported in neuro-psychological disorders such as schizophrenia (Moschopolous et al., 2019).

The ability to listen and communicate affects all aspects of life, and is isolating when lacking, potentially leading to loneliness, depression and unemployment. There is also increasing awareness that similar to hearing loss, impaired auditory processing leading to poorer speech in noise perception may increase the risk of dementia (Stevenson et al., 2022). Thus, establishing effective treatments for APD is vitally important.

### 1.4 Management of APD in adults

There is a growing body of literature that recognizes the importance of developing treatments for APD. However, most research is focused on the assessment and management of developmental APD. Adults with APD mainly fall into the acquired and secondary categories, with research into the management of adults with APD extremely sparse. Practice guidance is mainly based on anecdotal reports, case studies and research from other populations, highlighting the need for evidence-based research.

Current estimates of the prevalence of APD in the adult population in the UK vary widely and range from 0.9% of the total adult population (Hind et al., 2011) up to 76.4% of the over 55 year old population (Golding et al., 2004) which equates to between 485,000 and 15,945,000 adults potentially with APD and requiring access to effective management.

Current literature regarding interventions for adults with APD can be grouped into three main approaches: (1) modification of the environment, (2) auditory training and (3) compensatory strategies (British Society of Audiology, 2018).

#### 1.4.1 Modifying the listening environment

This approach, recommended by the BSA practice guidance (British Society of Audiology, 2018), involves improving signal clarity and reducing background noise. This bottom-up method (i.e., improving signal from the ear, up to the brain) has three approaches:

1) Adapting room to reduce reverberation together with noise reduction strategies.
2) Use of personal remote microphone systems (PRMS).
3) Use of low-gain hearing aids (LGHA), personal-sound-amplification-products (PSAPs) and “Hearables”.

##### 1.4.1.1 Adapting the room or workplace environment

For optimal speech intelligibility (in this review, “speech intelligibility” refers to the perception of speech), both acoustics and signal-to-noise ratio (SNR) need to be addressed. Both British Society of Audiology (2018) and the (American Academy of Audiology, 2010) recommend reducing background noise with soft furnishings, double glazing, acoustic wall paneling, ensuring the building conforms to acoustic regulations and finding optimal seating for the patient (ASHA, 2005b; Baran, 2014).

##### 1.4.1.2 PRMS

PRMS improve SNR and minimize acoustic signal distortion. Products range from analog frequency-modulated (FM) systems to more modern digital systems utilizing electromagnetic energy (Chisolm et al., 2007). The microphone transmitter, worn (or placed) by the speaker, is connected wirelessly to an in-ear receiver, sending the audio directly into the patient's ear which reduces the impact of reverberation, background noise and speaker distance, producing a clearer signal. This reduces cognitive effort so the listener may find complex listening situations less tiring. In addition to the immediate acoustic benefits of an increase in the SNR up to +25 dB (Crandell and Smaldino, 2000), longer term neuroplastic changes and psychosocial benefits have been reported (Keith and Purdy, 2014; Koohi et al., 2017b).

To date, the only systematic review of PRMS that included the adult APD population, was by Lemos et al. (2009). The inclusion criteria had no restrictions on age. Nineteen articles were included, most of them (70%) were classed as expert opinion, none were randomized controlled trials (RCTs) and none of the studies were based on adults. Hence, Lemos et al. (2009) concluded the use of PRMS in the APD population could not yet be recommended. Gallun et al. (2012), Saunders and Echt (2012), Gallun et al. (2017), and Tepe et al. (2020) have reviewed the use of PRMS as part of wider reviews into AP difficulties in the veteran populations, finding limited research in the target population. Research into PRMS use for APD management has occurred predominately in the pediatric population.

Following a systematic review into the effectiveness of PRMS for children with AP difficulties, Reynolds et al. (2016) concluded there was moderately strong evidence to suggest that PRMS were helpful in improving listening ability. However, children may not be able to utilize fully the sensory information, partly due to their incomplete linguistic/cognitive development. Thus, they are less able to “fill in” any missing or misheard words (Eisenberg et al., 2000). Any evidence of the effectiveness of PRMS in children cannot simply be extrapolated to the adult population.

PRMS are often used by adults with hearing loss (HL). In a systematic review Maidment et al. (2018) found improved speech intelligibility when using PRMS in conjunction with hearing aids (HAs) vs. HAs alone. AP difficulties often occur in the elderly alongside presbycusis, reducing HA benefit (Lesner, 2003). PRMS used in addition to HAs may be beneficial for the elderly APD population.

Practice guidance from American Academy of Audiology (2010), British Society of Audiology (2018), and American Speech-Language-Hearing Association (ASHA) (2005b) suggest using PRMS as an intervention for adults with APD. However, they all acknowledge the lack of firm evidence and clear need for further research in the adult APD population.

##### 1.4.1.3 LGHA, PSAPs and hearables

Traditionally only used for patients with HL, low level amplification has anecdotally been used as a treatment for patients with APD (Gallun et al., 2012; Atcherson et al., 2015). Although the improvement in SNR is likely to be inferior to that provided by a PRMS, there are distinct advantages as the speaker does not need to wear a microphone. For adults speaking to multiple people during a workday, conventional HAs may be more practical.

PSAPs and “Hearables” are potentially of interest with devices becoming cheaper and more sophisticated. A recent meta-analysis comparing PSAPs with conventional HAs in patients with HL (Chen et al., 2022) found any differences in speech intelligibility, sound quality and listening effort to be non-significant.

Currently the American Academy of Audiology (2010) and British Society of Audiology (2018) do not recommend LGHA as a treatment for APD. Conversely, the New Zealand guidelines *do* suggest that LGHA may be beneficial (Keith et al., 2019), however, supporting evidence came from a non peer-reviewed thesis involving eleven adults without an APD diagnosis (Moore, 2015). Evidence also suggests that binaural amplification may not always be acceptable by the elderly APD population with presbycusis, possibly preferring monaural HA use due to binaural interference (Holmes, 2003; Martin and Jerger, 2005; Atcherson et al., 2015).

To date, there have been no systematic reviews into LGHA use in the adult or pediatric APD population. Given the considerable uncertainty surrounding this intervention, further research is needed to help ascertain efficacy.

#### 1.4.2 Auditory training

Auditory training has been defined as a set of sound-related conditions and or tasks that are designed to activate auditory pathways to enhance the underlying neural activity and positively impact auditory behavior (Musiek et al., 2014). AT is considered a “bottom up” intervention as it involves improving the processing of the signal from the ear up to the auditory cortex. The delivery of this training can be formal, informal, clinic-based or home-based with the aid of computer-based auditory training (CBAT) programs and can involve verbal or non-verbal stimuli. All programmes involve repeated listening of a signal followed by a judgement regarding the signal, and then feedback on accuracy.

The BSA, AAA, and ASHA all recommend AT for adults with APD (ASHA, 2005a; American Academy of Audiology, 2010; British Society of Audiology, 2018). To individualize treatment, the type and focus of AT selected should depend on the type of deficits detected during the diagnostic process (American Academy of Audiology, 2010; Baran, 2014; British Society of Audiology, 2018). The training should be frequent, and appropriately challenging to optimize any neural changes (American Academy of Audiology, 2010). For rehabilitation to be effective, the adult brain needs to retain neuroplasticity. Numerous neurophysiological studies have indicated that AT causes changes in the neural connections in the adult brain (Kraus et al., 1995; Tremblay et al., 2001; Tremblay and Kraus, 2002; Kishon-Rabin et al., 2013; Li et al., 2018; Kawata et al., 2022). Whilst neural changes have been observed in adults, questions remain over the extent of change in behavioral aspects of auditory function caused by AT in an adult population with APD.

There have been several (non-systematic) reviews discussing efficacy of AT in the adult APD population (Gallun et al., 2012, 2017; Weihing et al., 2015; Tepe et al., 2020). All note the lack of research in adults and acknowledge that efficacy has yet to be determined.

Loo et al. (2010) systematically reviewed the pediatric APD literature focusing on the effectiveness of CBAT training, finding some evidence that Earobics^®^ and simple speech/non-speech sounds training improved AP indices, and weak evidence to support Fast ForWord^®^ training program due to mixed results and a lack of control groups. Fey et al. (2011) conducted a systematic review into auditory/language interventions for children with AP difficulties, finding weak evidence that AT improved AP. However, not all children were diagnosed with APD, so conclusions were unclear. The pediatric results whilst encouraging, are not directly comparable to adults due to differing brain plasticity, possibly requiring alternative stimuli and intensities of therapy.

Musical auditory training, when extended beyond passive listening, is thought to provide multiple benefits to the auditory system, in particular aiding auditory memory, auditory discrimination, temporal processing and speech-in-noise ability (Parbery-Clark et al., 2009; Strait and Kraus, 2011). It has been shown to enhance AP in musicians, with enhancements persisting into latter life, particularly for SIN test performance and gap detection thresholds (Zendel and Alain, 2012). No systematic reviews have included musical training as an intervention for the adult APD population. British Society of Audiology (2018) suggest musical training as potentially useful whilst acknowledging the lack of research.

#### 1.4.3 Compensatory strategies

These “top down” methods, utilizing cognitive processes to aid in the interpretation of the auditory signals, include meta-cognitive strategies such as; learning to self-regulate, using assertiveness to improve the listening environment and learning memory recall tactics. Meta-linguistic strategies such as developing knowledge of language to improve the ability to “fill” in any miss-heard words and improving listening ability, amongst others, are additionally used (Bamiou et al., 2006).

Most guidelines for APD (ASHA, 2005a; American Academy of Audiology, 2010; British Society of Audiology, 2018) advocate these strategies, although each acknowledges there appears to be little research on APD populations.

### 1.5 Gaps in knowledge

In the last 10 years, several reviews have been written on management strategies for adults with APD (Atcherson et al., 2015; Weihing et al., 2015), particularly in the veteran population with traumatic brain injury (Gallun et al., 2012, 2017; Saunders and Echt, 2012; Tepe et al., 2020). However, none of them followed a systematic search strategy. To the best of our knowledge, there is no current review following a systematic methodology, of the evidence surrounding the use of interventions that are recommended by international guidelines. Currently, due to a lack of research in the target population, the scientific basis behind recommendations has come from research in different populations such as children with APD and adults with HL. More recently interest in APD research has increased. There is now a need to evaluate the new data, thereby establishing a new evidence base from which to refocus research.

### 1.6 Aims

This review aims to systematically identify and critically evaluate evidence of the effectiveness of various interventions in treating adults with documented AP difficulties. Primary aims are to identify if there is any evidence that treatments are effective and establish the reliability of that data. Emphasizing areas that need further research and highlighting issues that are hindering progress in this field will be a secondary aim.

## 2 Methods

### 2.1 Eligibility criteria

Following the Preferred Reporting Items for Systematic reviews and Meta-Analyses (PRISMA) guidelines for the reporting of systematic reviews (Page et al., 2021), the inclusion criteria using the PICO (**p**opulation, **i**ntervention, **c**omparator, **o**utcome) framework are set out in Table 1.

**Table 1:** Inclusion and exclusion criteria.

**PICO**	**Inclusion criteria**	**Exclusion criteria**
Population	- Adults ≥ 18 years old - Participants with a confirmed diagnosis of APD OR with ≥ 1 abnormal result on a validated central auditory test^*^	- Participants with diagnosed cognitive disorders or unmedicated ADHD as these conditions can act as a confounding influence on auditory processing tests. - Participants with acute psychiatric conditions^**^,^***^ - Studies that involved adults AND children, unless the results were reported separately. - Studies involving patients with suspected APD - Any study involving participants with amusia, although a form of APD, it is diagnosed using different tests making comparisons difficult.
Intervention	- Any environmental modification was eligible as was any type of auditory training or compensatory strategy.	- Any study involving medication^***^ - Any study involving existing hearing aid users, as hearing aids are considered an intervention for APD and therefore would confound the results and not reveal the true effect of the intervention being studied.
Comparator	- Studies involving suitable control groups of any design - Studies with a repeated measures design with the pre-intervention measures used as a comparator	- Studies without any form of control such as case study reports. - Conference abstracts due to lack of study detail - Review papers, book chapters or expert opinions.
Outcomes	- Behavioral or electrophysiological tests sensitive to changes in the central auditory nervous system - Any validated questionnaire assessing hearing ability. No restriction on the duration of follow-up.	- Any measure not directly sensitive to changes in the central auditory nervous system.

^*^ ASHA (2005a) and American Academy of Audiology (2010) diagnosis criteria for APD requires performance at 2 SD below the mean on at least two AP tests, however the lack of worldwide agreement warranted a more relaxed inclusion criteria thereby not excluding potentially valid studies.

^**^Mechanism affecting CANS not fully understood, test results may be unreliable varying from day-to-day.

^***^Any medications involved may affect results on behavioral and/or electrophysiological tests. APD, auditory processing disorder.

### 2.2 Search strategy and study selection

Four of the most widely used databases in this field were searched, MEDLINE (Ovid), Embase (Ovid), Web of Science and Scopus. Three main concepts were identified from the review question: (a) APD (b) Adults (c) Intervention. For each concept, subject heading/MeSH (medical subject heading) terms and keywords were searched using all synonyms, truncating words and phrase mapping. There were no restrictions on language or year of publication, to endeavor to retrieve all relevant papers to the review question. All database searching was completed on 13th December 2023. After the duplicates were removed, the eligibility of papers was independently reviewed by two authors at each key step, including abstract screening and full-text reading. Lists of article selection by each author were compared for agreement. The controversial papers were further assessed by the third author for the final decision. The risk of bias of each individual study was assessed using seven bias domains of ROBINS-I tool (Sterne et al., 2016) as recommended in the Higgins et al. (2021). Each domain of the ROBINS-I tool is rated as Low, Moderate, Serious or Critical risk of bias, followed by an overall judgement that considers both the level of risk in each domain and the number of domains showing bias concerns. Any study deemed to be at “Critical” risk of bias was to be excluded from any meta-analysis (Sterne et al., 2021). The assessment was summarized visually using the Robvis tool (Mcguinness and Higgins, 2021).

### 2.3 Data synthesis and meta-analysis

Studies were systematically grouped according to the types of interventions and summarized into tables. Only groups of studies with similar design, intervention, and outcome were included in the meta-analysis which was conducted using Review Manager (REVMAN) (2020) software. The quality of the body of data for each outcome measure in the meta-analysis, was assessed using the GRADE approach (see Cochrane Handbook (Schünemann et al., 2021). Confidence in the quality and accuracy of the evidence is stated as “high,” “moderate,” “low,” or “very low.” As study sizes varied, the synthesis followed the generic inverse variance method, helping to create a more precise summary estimate with the larger studies given more weight (Deeks et al., 2021). As the study designs were varied, the random-effects analysis model was applied due to probable high heterogeneity (Reeves et al., 2021). Heterogeneity and its impact was analyzed using the *I*^2^ statistic [see The Cochrane Collaboration (Deeks et al., 2021)]. Values of between 0 and 40%, 30 and 60%, 50 and 90%, and 75 and 100% approximately show heterogeneity as being; not important, moderate, substantial and considerable, respectively.

## 3 Results

**Figure 1:**
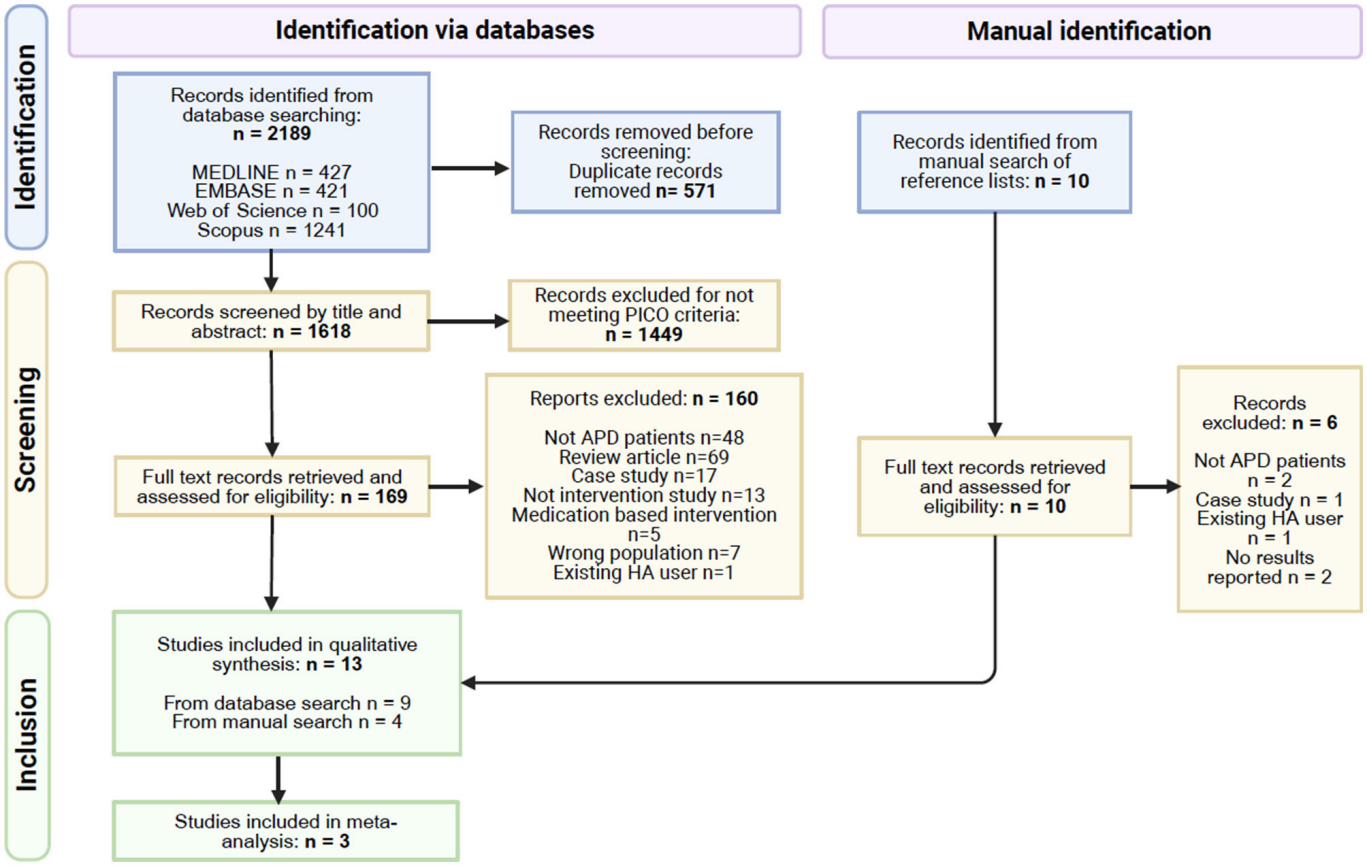
PRISMA flow diagram (Page et al., 2021), some studies had multiple reasons for exclusion.

**Figure 2:**
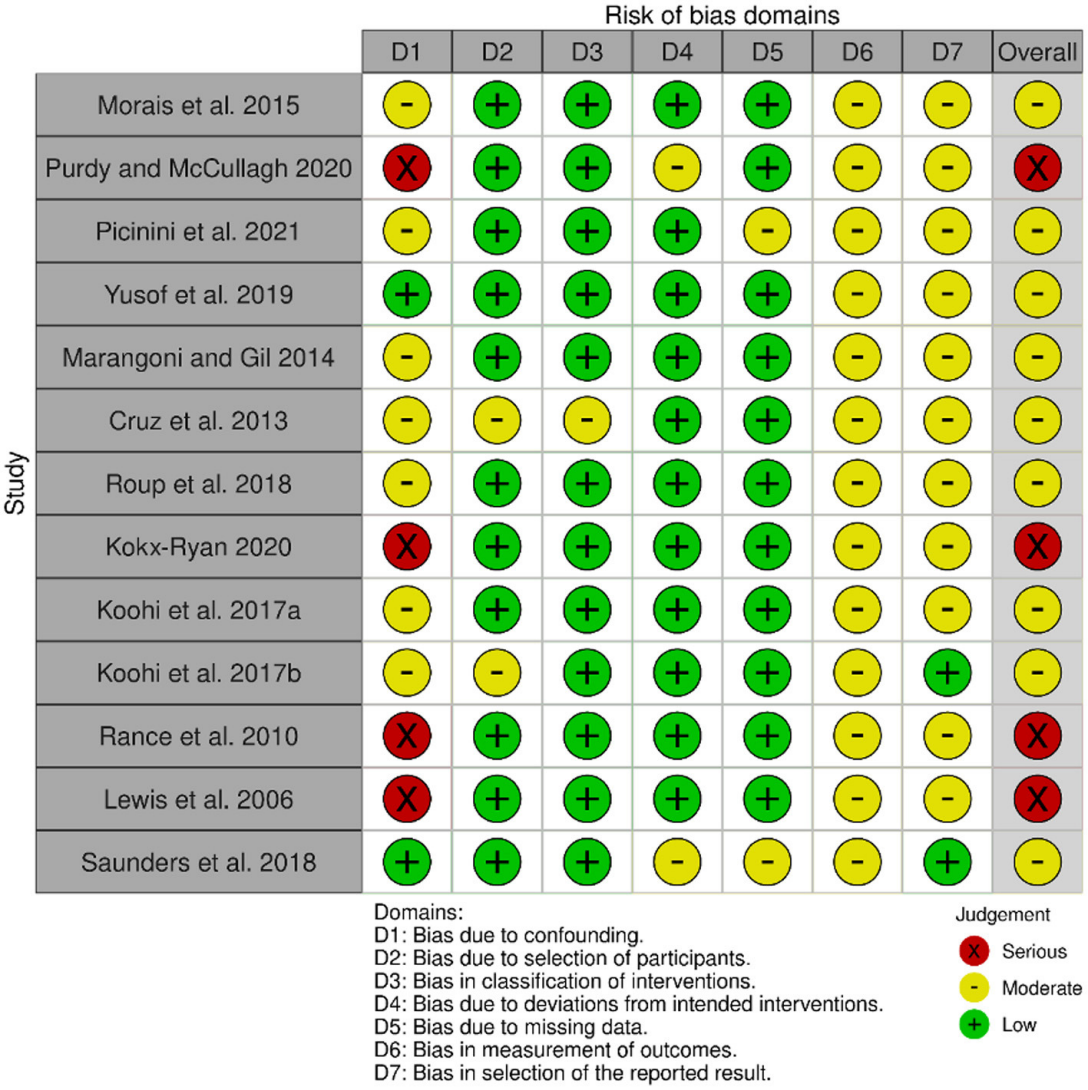
Risk of bias assessment using the ROBIN-I tool (Sterne et al., 2016), and the Robvis app (Mcguinness and Higgins, 2021).

The search retrieved 2,189 records, which after removal of duplicates left 1,618 records to be screened by title and abstract. Of 1,618, 1,449 records were excluded at this stage, leaving 169 records to move to the second stage of screening where full text was retrieved for all. Nine eligible studies were recruited to the review from this database search. The manual search of reference lists from relevant papers yielded ten records of interest which on closer examination of the full text led to the exclusion of six records. The remaining four records when added to the studies from the database search resulted in a total of 13 studies in the review. Figure 1 shows the PRISMA flow diagram for this selection process. The risk of bias for each study is summarized in Figure 2.

The thirteen studies were put in four groups according to the type of intervention investigated, either AT (*n* = 7), LGHA (*n* = 2), PRMS (*n* = 5) or PRMS, AT and standard care (*n*=1). The study by Saunders et al. (2018) was included in three groups due to multiple intervention arms. No studies were found to investigate compensatory strategies.

A variety of outcome measures were employed by the different studies and were grouped according to the auditory skills tested. As the focus of this review is on *effective* treatments for APD, two types of outcome measure were chosen for analysis and further discussion, (1) monaural low redundancy speech testing and (2) subjective listening ability. These measures determine “real life” benefits, not clinical tests to determine efficacy which do not necessarily reflect improvements in practical listening ability (Chermak et al., 2017).

### 3.1 Auditory training as an intervention for APD

Seven studies looked at the effect of AT, shown in Table 2. A variety of training methods were used; auditory skills-based training in clinic (Cruz et al., 2013; Marangoni and Gil, 2014; Morais et al., 2015), AT based on *a cappella* vocal duets (Picinini et al., 2021), novel computer-based auditory cognitive program (Yusof et al., 2019) and a home-based CBAT program (Saunders et al., 2018). The total hours of training varied from 6 to 40 h (see Table 2). Two studies used the same training program but differed substantially in study design, Cruz et al. (2013), was a retrospective study and Marangoni and Gil (2014), was prospective. The length of follow up varied across the studies from immediate, to up to 3 months after AT. Due to this heterogeneity, a meta-analysis was deemed inappropriate, as recommended in the Cochrane handbook (Reeves et al., 2021). The study by Purdy and Mccullagh (2020) did not use monaural low redundancy speech testing or subjective listening ability as outcome measures and therefore is not analyzed further in this review. This study showed some improvements in dichotic listening with inconsistent results.

**Table 2:** Characteristics of studies using Auditory Training as an intervention for APD.

**Study/design**	**Participants**	**PTA hearing thresholds**	**Intervention**		**Outcomes**	**Findings of study**	**Follow up**	**Compliance/attrition**
			**Type, frequency and duration**	**Home-based or clinic**				
Cruz et al. (2013)	APD patients ***N*** **=** **18** Nine Male Nine Female Age = 17–38 yrs	≤ 25 dBHL between 250 and 8,000 Hz	Formal AT, focusing on auditory closure, temporal processing, SIN ability, using verbal and non-verbal stimuli 45 min × 2 per week, total of eight sessions **Comparison**—Pre-AT baseline measures	In clinic	DPT FPT SSW SSI^*^	Significant improvements post AT in DPT and FPT (*p* < 0.001) for both genders, also for SSI (N/S −15) in females (*p < * 0.05*)* and SSW for males (*p* < 0.05).	Immediate	Retrospective
Retrospective study, repeated measures								
Marangoni and Gil (2014)	Patients with history of severe traumatic brain injury ***N*** **=** **9** Nine male Age = 20–37 yr	Within normal range between 250 and 8,000 Hz	Formal AT, focusing on auditory closure, temporal processing, SIN ability, using verbal and non-verbal stimuli 45 min × 2 per week, total of 8 sessions **Comparison**—Pre-AT baseline measures	In clinic	Sound localization SMVS SMNV SWN^*^ SSW SSI-ICM^*^ SSI-CCM^*^ DPT DCVT RGDT	Significant improvements after AT in: SMNV (four sounds), SSW, SSI-MCI, DPT (*p* < 0.05) Performance in RGDT and DCVT improved but not at a significant level	1 week after training finished	No mention of attrition.
Uncontrolled Before-After study								
Morais et al. (2015)	Older adults ***N*** **=** **16** 14 female Two male Age = 60–78 yr	< 40 dBHL at 500, 1,000, and 2,000 Hz	Acoustically controlled AT 50 min per week × 8 weeks. Verbal and non-verbal stimuli, focusing on auditory closure, temporal processing, SIN ability, cognitive ability, motor tasks Additional home-based training 15 min, 3 × week (Musiek and Schochat, 1998)	In clinic with home-based additional training	SIN^*^ DDT PPST GIN P300	A significant improvement was noted post AT in behavioral measures (*p* < 0.05) No significant improvements noted on P300, although authors note improved waveforms, latencies and amplitudes post training.	Retest at 4 weeks after AT	100% completed AT and assessments, no information on compliance of the home-based activities.
Controlled Before-After Study, crossover design			**Comparison**—Pre-AT baseline measures					
Picinini et al. (2021)	University students with APD ***N*** **=** **10** Three male Seven female Mean age = 23.4 ±3.8 yr	< 25 dBHL at 250–8,000 Hz.	Binaural AT using vocal duets, “Peixe Vivo” sung *a cappella* focusing on temporal processing and SIN ability 30 min × 2 per week. Between 6–10 h total. Three phases of challenge **Comparison**—Pre-AT baseline measures	In clinic	SIN^*^ FPT RGDT DCVT FFR ABR N2 P3	Significant improvement in SIN and FPT results following AT (*p* < 0.002). Authors note significant improvement at M3 in certain conditions for DCVT. Some evidence of auditory improvement from auditory evoked potentials at M3.	Immediate (M2) 3 months after AT (M3)	Two individuals were unable to attend M3
Uncontrolled Before-After study								
Purdy and Mccullagh (2020)	Stroke patients with aphasia ***N*** **=** **4** (one participant excluded as wears hearing aids) Four male Age = 31–64 yr	5–35 dBHL at 500, 1,000, and 2,000 Hz HF PTA 0–28 dBHL at 1,000, 2,000 & 4,000 Hz	Dichotic listening training 1 h, 3 × week for 6 weeks **Comparison**—Pre-AT baseline measures	In clinic	DDT Dichotic rhyme Competing sentences	Some improvements in dichotic listening were reported with variable results. No statistics performed. An improvement in the weaker ear often accompanied a weakening of the stronger ear.	Immediate	2 participants missed one session, 1 participant stopped after 14 sessions as he reached 70% accuracy,
Uncontrolled Before-After study								
Saunders et al. (2018)	Blast-exposed veterans ***N*** **=** **99** 88 male 11 female Age = 22–53 yr Four intervention arms: (1) CCS group, *n* = 25 (2) AT + CCS group, *n* = 25 (3) FM + CCS group, *n* = 25 (4) FM + AT + CCS group, *n* = 24	mean < 15 dBHL at 500–4,000 Hz	Trial for 8 week period CCS education- given three leaflets about auditory processing and communication strategies and discussed with audiologist. AT using Brain Fitness Program from Posit Science, focusing on temporal resolution, gap detection, sound discrimination, using verbal and non-verbal stimuli 1 h per day for 5 days a week. Intervention group–2 Comparison group–1	Computer based AT at home	HINT ATTR TCST^*^ SSQ-C^†^	No statistically significant improvements post AT (*p* > 0.05).	After 8 weeks of AT	Adherence to AT, 64.9% completing ≤ 10 sessions. 12 participants withdrew (36% attrition from group 2). Attrition group 1 0%
Randomized Controlled Trial								
ClinicalTrials.gov NCT00930774								
Yusof et al. (2019)	Older adults ***N*** **=** **43** *AT group n* = 23 7 Male 16 Female Mean age = 67.6 ± 4.5 yr *Control group n* = 20 3 Male 17 Female Mean age = 65.8 ± 3.6 yr	4 freq. average 500–4,000 Hz ≤ 30 dBHL	Treatment group (*n* = 23): Computer-based auditory-cognitive training. Focusing on SIN ability, working memory, ability to reduce cognitive interference, using verbal stimuli 45 min, 3 × per week for 8 weeks Control group (*n* = 20): documentary videos same frequency and duration as treatment group **Comparison**—control group	Supervised at a day center	MyHINT (HINT [quiet], HINT[composite])^*^ Malay DDT GIN PPST	Significant improvements seen in Malay DDT, GIN, MyHINT (quiet), and PPST (humming) (*p* < 0.05) after training vs. control group. No significant changes in results after 4 weeks post AT, suggesting AT benefits are maintained for at least 1 month. No significant improvements in HINT (composite) or PPST (verbal) test.	After 4 and 8 weeks of AT and 4 weeks post training.	100% adherence to training
Randomized controlled study								

^*^Outcome measures for monoaural low redundancy speech testing.

^†^Outcome measure for subjective listening ability.

KEYS: AT, auditory training; SIN, speech-in-noise; DDT, Dichotic Digit Test; PPST, Pitch Pattern Sequence Test; GIN, Gaps-In-Noise test; HF PTA, high frequency pure tone average; APD, Auditory Processing Disorder; RGDT, Random Gap Detection Test; DCVT, dichotic consonant vowel test; FFR, frequency following response; FPT, Frequency Pattern Test; ABR, Auditory Brainstem Response; MyHINT, Malay Hearing-In-Noise Test; SMVS, sequential memory of verbal sounds; SMNV, sequential memory of non-verbal sounds; SWN, speech with white noise; SSW, Staggered Spondaic Word test; SSI-ICM, synthetic sentence identification with ipsilateral competing message; SSI-CCM, synthetic sentence identification with contralateral competing message; DPT, Duration Pattern Test; N/S, noise-to-signal ratio; ATTR, Adaptive Tests of Temporal Resolution; TCST, Time-Compressed Speech Test; SSQ-C, Speech Spatial Qualities questionnaire-comparative version; CCS, compensatory communication strategies; FM, frequency-modulated system.

#### 3.1.1 Monaural low redundancy speech testing

Six studies (Cruz et al., 2013; Marangoni and Gil, 2014; Morais et al., 2015; Saunders et al., 2018; Yusof et al., 2019; Picinini et al., 2021) used monoaural low redundancy speech testing with different outcome measures (see Table 2). Four of six studies showed significant improvement of speech test performance (*p* < 0.05), except the studies from Saunders et al. (2018) and Yusof et al. (2019), where authors concluded marginal improvements were due to possible test-retest effects.

#### 3.1.2 Subjective listening ability

Saunders et al. (2018) was the only study to measure subjective listening ability following AT. The authors noted no significant improvements following AT (*p* > 0.05), when compared to the control group.

### 3.2 LGHA as an intervention for APD

Two studies investigated the effect of LGHA and the main characteristics are shown in Table 3. Similarities exist between the populations; blast-exposed veterans with mild TBI (Kokx-Ryan, 2020) and participants with self-reported hearing difficulties of which 47% with probable TBI (Roup et al., 2018). Both studies used receiver-in-the-canal (RIC) aids worn bilaterally with open domes. Meta-analysis was not conducted due to the limited number of studies involved.

**Table 3:** Characteristics of studies using LGHA as an intervention for APD.

**Study/design**	**Participants**	**PTA hearing thresholds**	**Intervention**		**Outcomes**	**Findings of study**	**Follow up**	**Compliance/attrition**
			**Type, frequency and duration**	**Home-based or in clinic?**				
Kokx-Ryan (2020)	Blast-exposed veterans with diagnosis of acquired APD, confirmed mild traumatic brain injury ***N*** **=** **15** 18–55 yr	≤ 25 dBHL 250–8,000 Hz	Low-gain hearing aids (ReSound Linx Quattro TS61 RIC or Phonak Audeo Marvel 90 RIC, open domes) use for 2 weeks. **Comparison**—unaided baseline measures	Hearing aid use during clinical stay at medical center	APHAB QuickSIN_s_ mQuickSIN_s_	Significant auditory improvements shown on APHAB (*p* < 0.001), QuickSIN *(p* < 0.001)_s_ and mQuickSIN (*p* < 0.001)s when aided.	After 2 weeks of hearing aid use.	Compliance average of 12 h a day.
Uncontrolled Before-After study								
Roup et al. (2018)	Adults with self-reported hearing difficulties ***N*** **=** **19** 2 withdrew leaving 17 5 Male 12 Female 18–58 yr	≤ 25 dBHL 250–8,000 Hz	Low-gain hearing aids (Widex Dream 440 Fusion, RIC, open domes) worn for a min 4 h per day for 4 weeks **Comparison**—unaided baseline measures	Hearing aid use at home	HHIA APQ R-SPIN (at four different SNRs)	Significant reduction in hearing difficulties when aided vs. unaided (HHIA, *p* < 0.05; APQ, *p* < 0.05) Significant improvement on R-SPIN for high and low predictability sentences and at all SNRs when aided compared to unaided (*p* < 0.05).	Baseline and after 4 weeks of hearing aid use.	Hearing aids worn for a variable amount of time, two participants withdrew
Uncontrolled Before-After study								

PTA, pure tone average; RIC, receiver-in-canal hearing aids; APHAB, Abbreviated Profile of Hearing Aid Benefit; QuickSIN_s_, Quick Speech-In-Noise test soundfield; _m_QuickSIN_s_, Modified Quick Speech-In-Noise test soundfield; HHIA, Hearing Handicap Inventory for Adults; APQ, auditory processing questionnaire; R-SPIN, Revised Speech Perception-In-Noise test; SNR, signal-to-noise ratio.

#### 3.2.1 Monaural low redundancy speech testing

Both the Roup et al. (2018) and Kokx-Ryan (2020) studies used monaural low redundancy speech testing with different outcome measures (see Table 3). Both studies showed improvements in speech intelligibility when participants were aided (*p* < 0.05).

#### 3.2.2 Subjective listening ability

Questionnaires were used to assess listening ability in the unaided and aided condition. Results from both studies (Roup et al., 2018; Kokx-Ryan, 2020) showed overall improvements in subjective listening ability when participants were aided (*p* < 0.05).

### 3.3 PRMS as an intervention for APD

The PRMS used in all five included studies were personal FM systems, intended for patients with near-normal thresholds. Table 4 showed the main characteristics of those studies. Underlying health conditions of the participants and duration of FM system use varied for each study.

**Table 4:** Characteristics of studies using PRMS as an intervention for APD.

**Study/design**	**Participants**	**PTA hearing thresholds**	**Intervention**		**Outcomes**	**Findings of study**	**Follow up**	**Compliance/attrition**
			**Type, frequency and duration**	**Home-based or in clinic?**				
Koohi et al. (2017a)	Stroke patients ***N*** **=** **9** 8 Male 1 Female 24–78 yr	< 25 dBHL 500–8,000 Hz	Binaural FM system (Phonak iSense Micro receiver, zoomLink+ transmitter) **Comparison**—Unaided baseline measures	FM use only in clinic	BKB sentences with multi-talker babble noise presented at 0° and ±90° azimuth, SRM calculated	Significant increase in SRM when aided *and* the noise and signal are spatially separated (*p* = 0.0000). Cohen's *d* showed a large effect size (*d* = 0.93)	Immediate	Immediate therefore not applicable
Uncontrolled Before-After study								
Koohi et al. (2017b)	Stroke patients ***N*** **=** **9** 8 male 1 female 24–78 yr	< 25 dBHL 500–8,000 Hz	Intervention group (*n* = 4) Binaural FM system use for 10 weeks (Phonak iSense Micro receiver, zoomLink+ transmitter). Control group (*n* = 5) Received standard care. **Comparison**—control group	FM use at home	BKB sentences with multi-talker babble noise presented at 0° and ±90° azimuth	Significant (*p* < 0.05) improvements were reported, after 10 weeks of FM use, on the speech-in-noise test in the aided *and* unaided conditions when noise presented from the left in the intervention group compared to control group.	Baseline and after 10 weeks use.	Self-reported use of FM system minimum 4 h per day.
Prospective Non-randomized controlled trial								
Lewis et al. (2006)	Multiple Sclerosis patients ***N*** **=** **10** 6 Male 4 Female Mean age = 50 ± 6 yr	< 20 dBHL 250–2,000 Hz < 40 dBHL 4,000–8,000 Hz	Phonic Ear PE 300T FM transmitter. Phonic Ear PE 300R FM receiver, with a boom microphone **Comparison**—Unaided baseline measures	In clinic	Speech-Perception in noise IEEE sentence list- Multitalker babble	The Multiple Sclerosis group performed significantly better in the aided condition than the unaided (*p* < 0.01). Authors note, not everyone benefitted.	Immediate	Immediate therefore not applicable
Uncontrolled Before-After study								
Rance et al. (2010)	Friedreich Ataxia patients ***N*** **=10** 8–42 yrs (6 adults 4 children) Six male adults	4 freq. average ≤ 30 dBHL	Phonak Inspiro FM transmitter with bilateral iSense receivers, used for 6 weeks **Comparison**—Unaided baseline measures	FM use at home/work	APHAB Speech perception testing with consonant-nucleus-consonant list with noise	Speech perception was significantly improved in the aided condition (*p < *0.05) The APHAB data for adults and children was not reported separately.	Speech testing: immediate. APHAB: Baseline, 2 + 4 weeks of use, and after 2 weeks of non-use.	Self-reported use of FM system 1–5 h per day
Uncontrolled Before-After study								
Saunders et al. (2018)	Blast-exposed veterans ***N*** **=** **99** 88 Male 11 Female Age = 22–53 yr, Four intervention arms: (1) CCS group *n* = 25 (2) AT + CCS group *n* = 25 (3) FM + CCS group *n* = 25 (4) FM + AT + CCS group *n* = 24	< 15 dB HL 500–4,000 Hz	Trial for 8 week period: CCS education- given three leaflets about auditory processing and communication strategies and discussed with audiologist. FM systems used were bilateral Phonak iSense Micro Dynamic FM receiver, Zoom Link+ Dynamic Transmitter. Intervention group–3 Comparison group—1	FM use at home	HINT ATTR TCST SSQ-C	Significant improvements in SRT post intervention for participants in the groups using FM systems (*p* < 0.001). Significantly higher scores on the SSQ-C Speech and Qualities scales (*p* < 0.05) post intervention compared to the groups without FM systems. No significant improvements in ATTR or TCST measures.	After 8 weeks use	Adherence to FM system. 82.2% using FM system for 2/3 days a week Attrition group 1 0% Attrition group 3 4%
Randomized Controlled Trial								
ClinicalTrials.gov NCT00930774								

PTA, pure tone average; FM, frequency modulated; BKB, Bamford-Kowal-Bench; SRM, Spatial release from masking; APHAB, Abbreviated Profile of Hearing Aid Benefit; CCS, compensatory communication strategies; AT, auditory training; HINT, Hearing-in-noise test; ATTR, Adaptive tests of temporal resolution; TCST, Time-compressed speech test; SSQ-C, Speech Spatial Qualities questionnaire-comparative version; SRT, Speech reception threshold.

#### 3.3.1 Monaural low redundancy speech testing

**Figure 3:**
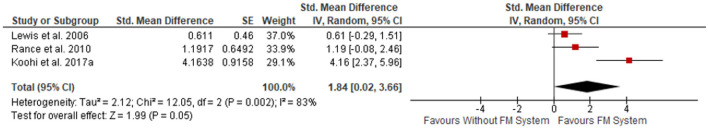
Meta-analysis of monaural low redundancy speech testing results, with FM system vs. without FM system, SMD plotted with 95% CI, in order of increasing effect size [Review Manager (REVMAN), 2020].

All five studies used these outcome measures and reported improvements in speech intelligibility when using FM systems compared to without. Differences in study design resulted in the exclusion of Koohi et al. (2017b) and Saunders et al. (2018) from the meta-analysis. Note the Koohi et al. (2017a,b) studies involved the same participants. Meta-analysis was conducted on the three studies with similar (uncontrolled before-after) design (Figure 3). There was a significantly better performance in the FM system group, with a standardized mean difference (SMD) of 1.84 (95% CI, 0.02–3.66). However, the heterogeneity was substantial (*I*^2^ = 83%). GRADE assessment revealed a “Low” certainty of evidence (GRADE assessment shown in Appendix A).

Koohi et al. (2017b) investigated the long-term effect of FM system use, finding significant improvements in speech reception threshold in the unaided and aided condition (when noise was coming from the left) following 10 weeks of FM system use when compared to a control group only using the FM system for testing (*p* < 0.05). Thus, providing evidence for the possible neural changes this technology may bring beyond the immediate benefits to speech intelligibility.

#### 3.3.2 Subjective listening ability

Two studies used questionnaires to assess listening ability in the unaided and aided condition (Rance et al., 2010; Saunders et al., 2018). Rance et al. (2010) presented combined adult and child APHAB results therefore cannot be analyzed further, although the mean data for all participants showed an improvement in subjective listening ability when aided compared to unaided (*p* < 0.05). Saunders et al. (2018) observed an improvement in subjective listening ability (using the SSQ-C questionnaire) when aided compared to unaided (*p* < 0.05).

### 3.4 Use of PRMS in conjunction with AT and standard care, as an intervention for APD

One study (Saunders et al., 2018) looked at the effect of combining; FM system use, regular AT and standard care [compensatory communication strategies (CCS)], comparing the results to the standard care group and groups using the FM systems and AT separately. Table 5 shows the main characteristics of this study.

**Table 5:** Characteristics of study using PRMS in conjunction with AT and standard care, as an intervention for APD.

**Study/design**	**Participants**	**PTA hearing thresholds**	**Intervention**		**Outcomes**	**Findings of study**	**Follow up**	**Compliance/attrition**
			**Type, frequency and duration**	**Home-based or in clinic?**				
Saunders et al. (2018)	Blast-exposed veterans ***N*** **=** **99** 88 Male 11 Female Age = 22–53 yr, Four intervention arms: (1) CCS group *n* = 25 (2) AT + CCS group, *n* = 25 (3) FM + CCS group, *n* = 25 (4) FM + AT + CCS group, *n* = 24	Mean < 15 dBHL at 500–4,000 Hz	Trial for 8 week period CCS education- given three leaflets about auditory processing and communication strategies and discussed with audiologist. AT using “Brain Fitness Program from Posit Science” 1 h per day for 5 days a week FM systems used were bilateral Phonak iSense Micro Dynamic FM receiver, Zoom Link+ Dynamic Transmitter. Intervention group–4 Comparison group–1	Computer based AT at home FM use at home	HINT ATTR TCST SSQ-C	Significant improvements in SRT (measured by the HINT test) post intervention for participants in the groups using FM systems (*p* < 0.001). Significantly higher scores on the SSQ-C Speech and Qualities scales (*p* < 0.05) in FM + CCS group and FM + AT + CCS group compared to the groups without FM systems. No significant improvements in ATTR or TCST measures. AT not found to improve SRT.	After 8 weeks of intervention	Adherence to AT, 64.9% completing ≤ 10 sessions. Adherence to FM systems. 82.2% using FM system for 2/3 days a week Attrition in group 1, 0% Attrition in group 4 8.3%
Randomized controlled trial								
ClinicalTrials.gov NCT00930774								

PTA, pure tone average; FM, frequency modulated; CCS, compensatory communication strategies; AT, auditory training; HINT, Hearing-in-noise test; ATTR, Adaptive tests of temporal resolution; TCST, Time-compressed speech test; SSQ-C, Speech Spatial Qualities questionnaire-comparative version; SRT, Speech reception threshold.

#### 3.4.1 Monaural low redundancy speech testing

The study from Saunders et al. (2018) showed that the groups using FM systems (FM + CCS group and FM + AT + CCS group) significantly improved performance in HINT following 8 weeks of intervention (*p* < 0.001), compared to the two other group (AT + CCS group and CCS alone group). However, there was no obvious difference in performance between the FM + CCS group and FM + AT + CCS group. Authors concluded that improvements were due to FM system use; in contrast, AT bringing no further benefit to speech intelligibility although the rate of attrition and adherence was higher in the AT groups.

#### 3.4.2 Subjective listening ability

Saunders et al. (2018) reported the significantly better SSQ-C score in the FM + CCS group and FM + AT + CCS group, compared to AT + CCS group and the group that used CCS alone (*p* < 0.05). Therefore, using FM system in combination with other methods could be beneficial to listening ability.

## 4 Discussion

Thirteen studies met the inclusion criteria and were grouped according to intervention category. A variety of outcome measures were examined and mainly grouped into (1) monaural low redundancy testing and (2) subjective listening ability. Only one study (Purdy and Mccullagh, 2020) did not use these two outcome measures, therefore it is not included in the discussion.

### 4.1 Effectiveness of AT as an intervention for adults with APD

Four of the six studies using speech testing (Cruz et al., 2013; Marangoni and Gil, 2014; Morais et al., 2015; Picinini et al., 2021) found evidence of an intervention effect with significant improvements in speech test performance after AT.

Two studies (Saunders et al., 2018; Yusof et al., 2019) reported no significant improvements of speech intelligibility in noise after AT. Yusof et al. (2019) reported significant improvement in performance of Dichotic Digit Test (DDT) (which is a test of binaural speech integration) after AT. Both studies (Saunders et al., 2018; Yusof et al., 2019) were of a stronger methodological design than the previously mentioned studies, both being RCTs and having larger sample sizes, although both studies were still considered at moderate risk of bias. In addition, although the study by Saunders et al. (2018) was of a fairly robust design, there was low adherence to the training, with 64.9% of participants only completing up to a quarter of the recommended home-based sessions. Low compliance rates make it difficult to draw conclusions regarding the efficacy of the “Brain Fitness Program”. Compliance of home based training has been shown to be an issue previously (Sweetow, 2009) and a previous review on the veteran population also commented on the time intensive nature of AT not being very practical for busy middle aged veterans (Gallun et al., 2017). These drawbacks need to be considered when designing CBAT programmes.

Similarly, the results of previous reviews of CBAT are mixed. In the pediatric APD population Loo et al. (2010) concluded that although benefits were seen in some studies there was often a lack of control groups and small study sizes. In a systematic review of the benefit of CBAT in the adult HL population, Henshaw and Ferguson (2013) found weak evidence of improvements in speech intelligibility following training, though there was large variability within, and between, the studies.

From the research gathered in this review it is impossible to determine which type of AT is best for each area of auditory deficit. Only one study, Morais et al. (2015), used deficit specific training. However, all participants (and data) were grouped together, therefore analysis of the effect of training on each area of deficit is not possible. In a review on the use of AT in the APD population, Weihing et al. (2015) noted that training often took a wide battery approach, making conclusions about training for specific auditory areas difficult. This current review shows research has not progressed in this regard since then.

Longer term improvements in speech intelligibility following AT was detected in one study (Picinini et al., 2021). Picinini et al. (2021) suggested that improvements in speech intelligibility may be maintained for up to 12 weeks post AT, though the evidence was weak due to a lack of control group in their cohort. In a systematic review of CBAT (Henshaw and Ferguson, 2013), it was reported that improvements were maintained up to 7 months post training in patients with HL. Although in a review by Loo et al. (2010) longer term benefits up to 12 months were inconclusive.

In addition to concerns over the quality of studies in this review, comparing them was challenging because of a large heterogeneity between the studies, including the:

- Varied APD populations (elderly participants, aphasia patients, blast exposed veterans, university students with APD and patients with TBI).
- AT material (formal and computer based); location of training (clinic or home based); length of training, and auditory skill being trained.
- Large variety of speech testing used as outcome measures, with differences in method, stimuli and recorded units.
- Peripheral hearing level in some studies were within normal limits whilst others permitted up to 40 dBHL. With some AP tests sensitive to hearing loss, particularly speech testing, there is the potential for HL to make results unreliable.

Many of the above issues have been highlighted in a recent rapid review by Gaeta et al. (2021) of AT interventions for adults with HL, they found methodological concerns within the included studies a possible hindrance to determining efficacy.

Weihing et al. (2015) noted that increased training effects were seen when the AT and testing material were similar (near-transfer of learning). For AT to be classed as *effective*, far-transfer of learning needs to occur, where trained tasks lead to generalized improvements in functional listening. As yet, there is not the evidence, from high quality studies, to confirm efficacy or effectiveness of AT in adults with APD when measured by *functional* outcome measures. However, this review does provide limited evidence that supervised AT (formal auditory skills-based or vocal duet training), may be an active treatment for APD in adults.

### 4.2 Effectiveness of LGHA as an intervention for adults with APD

The evidence from the two studies in this review (Roup et al., 2018; Kokx-Ryan, 2020) suggests that open fit, LGHA, with directional microphones and noise reduction capabilities may improve speech intelligibility, when assessed by speech testing or subjective listening ability, in adults with probable TBI and APD.

Unlike Kokx-Ryan (2020) and Roup et al. (2018) reported that benefits were not universal, noting that participants with a score of ≥34 on the HHIA were more likely to gain benefit from LGHA. These finding are of interest because the use of LGHA is not recommended by existing guidelines for the APD population, with the exception of New Zealand. The two studies in this review are the first to be performed on adults. Only one study using LGHA (mild-gain, open-ear fitting hearing aids with a directional microphone and noise reduction algorithm) has been conducted in the pediatric APD population (Kuk et al., 2008), and although improvements were reported in speech intelligibility in noise (when directional microphone or noise reduction program were enabled), improvements were highly variable and non-significant unlike the studies in this review. However, the use of LGHA in the normal threshold pediatric population may be less successful than in adults due to the classroom environment. The teacher may be further than two meters away from the child, outside the optimal distance for HA microphones to detect the speech signal.

Whilst the results of a study involving participants with mild TBI (Kokx-Ryan, 2020) and one where 47% of participants had a history of probable TBI (Roup et al., 2018), reflect the potential benefits perceived by that specific population, it is not possible to extrapolate the findings to the whole APD adult population.

Even though the results show positive improvements in speech-in-noise ability, only 18% of participants with hearing difficulties in the Roup et al. (2018) study purchased HAs immediately following the trial, possibly indicating the “real world” benefits were less noticeable. Similarly in a recent study (Singh and Doherty, 2020) involving the use of LGHA by adults with self-reported hearing difficulties and normal hearing thresholds, despite improvements in speech intelligibility when aided, only 20% wished to purchase the hearing aids after the trial. LGHA can increase SNRs, however, such increases should be noticeable, meaningful, or important to patients. Even with noticeable clinical improvements LGHA may not suit everyone. McSheerty et al. (2016) highlight the difference between what is a noticeable and what is a meaningful difference in SNR. In their cohort, although the participants were able to detect differences in SNR of 3 dB (noticeable difference), they consider intervention only when differences in SNR reached at least 6 dB (just meaningful difference). They concluded that noticeability, meaningfulness, and importance need be carefully distinguished.

Both the Roup et al. (2018) and Kokx-Ryan (2020) studies lacked appropriate age matched control groups so placebo effects cannot be ruled out, particularly in the subjective listening ability measures. In addition, the study by Kokx-Ryan (2020) was judged at “serious” risk of bias due to confounding factors and also was a doctoral thesis and therefore lacked the rigorous scrutiny of the peer review process. Due to the paucity of data regarding efficacy in this population, further research with high quality study designs is desperately required.

### 4.3 Effectiveness of PRMS as an intervention for adults with APD

The results of the meta-analysis provide supporting evidence that speech intelligibility is improved when using a PRMS compared to without, in patients with AP difficulties. However, studies in the meta-analysis had different co-morbidities within the population, raising the possibility that the results from each study may be more indicative of the benefits of PRMS for that particular APD population rather than generalizing the benefits to all APD patients.

Benefit varied within studies. Rance et al. (2010) found all adult Friedreich's Ataxia patients improved, but only 67% significantly improved, although some patients were in the advanced stages of this progressive disorder. Lewis et al. (2006) reported 20% of MS patients did not receive benefit at certain SNR. Saunders et al. (2014) reported in a separate paper from the RCT (Saunders et al., 2018), the subjective outcomes of three participants in the FM trial, revealing that one of the three did not perceive benefit. In contrast, 100% of stroke patients in the FM trial significantly improved their speech intelligibility in noise when aided compared to unaided (Koohi et al., 2017a,b). Such population differences make generalizing the reasons behind the variation in performance difficult. Saunders et al. (2018) noted that those with the poorest SIN results generally perceived most benefit. Lifestyle was extremely important, with more benefits felt for those who socialized regularly, than for patients living in a quiet environment. Concerns over aesthetics were also a potential barrier to successful PRMS use.

Possible long term neuroplastic changes were detected in one study in this review (Koohi et al., 2017b). Previous evidence for neuroplastic changes from FM use have been noted in a review by Keith and Purdy (2014), who reported improvements in speech intelligibility, in the unaided condition for children with APD following FM system use. In contrast, a recent RCT looking at remote microphone hearing aid use amongst children with APD (Stavrinos et al., 2020) found no significant improvements in the unaided listening-in-noise ability after 6 months of use, although it was noted that the baseline performance was already within normal limits reducing the capacity for improvement.

The results of this meta-analysis must be viewed with caution as the 95% CIs were wide, suggesting low confidence in the precision of the effect size, along with a high value for the heterogeneity. In addition to population differences, outcome measures were varied in method, stimuli and units making comparisons challenging. Only three studies were incorporated in the summary statistics, all were lacking control groups. In addition, two of the three studies (Lewis et al., 2006; Rance et al., 2010) were judged at “serious” risk of bias due to poor study design and uncontrolled confounding factors, which contributed to the “low” certainty of evidence provided by the GRADE assessment. However, two controlled studies not included in the meta-analysis (Koohi et al., 2017b; Saunders et al., 2018) add weight to the evidence of the benefits of PRMS to this population, and so despite discussed drawbacks this review provides low to moderate evidence supporting the use of PRMS with more research needed to establish the full intervention effects.

### 4.4 Effectiveness of PRMS in conjunction with AT and standard care as an intervention for adults with APD

One study in this review combined interventions (Saunders et al., 2018), reporting that combining AT, FM system and standard care provided no more benefit to speech intelligibility and subjective listening ability than the FM system and standard care. Although the AT program was not well-adhered to, on average < 25% of the training sessions were completed in the FM, AT and standard care group. The authors also note adherence to interventions was better when fewer interventions were combined. This study involved blast-exposed veterans and so any findings cannot be extrapolated to the wider APD population.

Similarly, inconclusive results were obtained in an RCT examining the effect of combining FM system use with either discrimination training or language training on 55 children diagnosed with APD (Sharma et al., 2012). No improvement in speech intelligibility (measured by HINT testing) was noted following intervention in any treatment group. Mixed results for other outcome measures in the study reveal an unclear picture of the benefit or otherwise of combining interventions. The groups with FM systems appear to be unaided when testing so immediate benefits due to FM systems are not recorded, although neurological changes due to FM systems seem to be absent. Caution is needed when comparisons are made to the pediatric population as developmental APD may require different treatments to adults with acquired APD. With only one study combining interventions on adults with APD (Saunders et al., 2018), there is not enough evidence to draw firm conclusions, therefore, further research is needed to explore the effects of combining interventions on this population.

### 4.5 Limitations of this systematic review

#### 4.5.1 Limitations in data collection

APD testing protocols vary across the world with “abnormal” criteria ranging from 1 to 2 SD below the mean, introducing the risk that participants have been included in this review who do not have APD. Due to the paucity of research it was felt this laxer approach was necessary so not to exclude relevant research, but it does, nevertheless, reduce the validity of the conclusions.

The search was unrestricted by year, language and outcome measure to increase the chances of collecting all relevant papers. However, unpublished reports and gray literature were not systematically searched beyond the four databases. Conference abstracts were not included due to the lack of detail presented. Therefore, the review is susceptible to publication bias, with non-significant results less likely to be reported (Hopewell et al., 2009). In a field of limited published research, the impact of missing unreported research could be large, and result in over-estimating the benefits of intervention. Despite an extensive database search, four of the studies in the review were found by reference searching, clearly missed by the database search, which raises concerns that other records have been missed.

#### 4.5.2 Limitations in analysis

Small scale studies, such as the ones in this review, are more likely to show large effect sizes and be more imprecise (Boutron et al., 2022), therefore should be interpreted with caution. Reporting results using SMD, although necessary when there is heterogeneity in outcome measures and equipment, has limitations as the interpretation of the effect size is generic across all research (Cohen, 1988; Sawilowsky, 2009) and is not linked to the impact on the patients. Reducing the heterogeneity between studies would allow the use of mean difference (MD) values and lead to more meaningful interpretation of results and understanding of the impact of interventions. Data was extracted from the different studies before *post-hoc* analyses in order to compare pre/post measures with other studies. However, this reduces the accuracy of the effect size as confounding factors have not been controlled for.

### 4.6 Study implications and directions for future research

This is the first systematic review and meta-analysis of the effectiveness of interventions for the adult APD population. Despite the limitations just discussed, and the limitations within the included studies, this review extends previous literature by providing a systematic and contemporary assessment of all intervention studies to date, thus providing evidence-based conclusions on the current position regarding effectiveness of these treatments for this population. From this review, recommendations (outlined below) can be identified for future research, which if implemented, may enable efficacy and then effectiveness to be ascertained, in the hope that clinicians of the future will be able to confidently recommend evidenced-based treatments for their patients.

Firstly, research needs to focus on developing and adopting a “gold standard” method of diagnosis/diagnostic criteria. This is needed to reduce the heterogeneity of the population in the body of research. Secondly as APD is a heterogenous disorder, future studies need to carefully characterize the patients with AP deficits and other assessments such as cognitive tests should be used and controlled for when assessing intervention, to determine the intervention effect on that area of AP. Although challenging to achieve, ideally the incorporation of an age, gender, handedness, APD deficit matched control group within a randomized study design, would enable efficacy to be firmly established. Studies also need to include longer follow-up times to examine the long-term impact of treatment and there needs to be a standardization of auditory training techniques and stimuli to allow more meaningful comparisons of these interventions.

Currently, a variety of outcome measures complicates and impedes the comparison of studies. Further research is needed to establish which outcome measures best identify changes in the CANS and, more importantly, that reflect meaningful improvements in day-to-day life, with the ultimate goal of intervention to improve patient satisfaction in their auditory life, not to improve on clinical tests. PSAPs, “Hearables” and smart phone apps are being marketed at APD populations (Nuheara, 2021), however, there appears to be no independent peer-reviewed research on the use of these devices in the adult APD population. Future research needs to be conducted to examine the efficacy of these new devices.

## 5 Conclusion

In summary:

- **AT:** Evidence that AT improves speech intelligibility in adults with APD remains mixed, with supervised training likely to be more successful than home-based, from evidence of low to moderate quality.
- **LGHA:** Low quality and limited evidence support the use of low-gain hearing aids, to increase speech intelligibility for adults with APD and a history of TBI.
- **PRMS:** Some low to moderate quality evidence supporting the efficacy of PRMS for increasing speech intelligibility in adults with APD when aided, with possible evidence of long term neuroplastic changes in the unaided listening condition.
- **Combination of interventions:** No evidence combining interventions improves speech intelligibility beyond the use of PRMS alone. Although evidence only from one study.

This review has indicated an improvement in the quality of evidence, transitioning from the anecdotal evidence and professional opinion of the past, as noted in previous reviews (Bamiou et al., 2006; Lemos et al., 2009; Gallun et al., 2012; Atcherson et al., 2015), toward intervention studies involving a diagnosed APD population. However, these results do not provide sufficient evidence to prove effectiveness, with the main weakness being poor, low-powered study designs lacking controls.

Our systematic review aimed to synthesize current evidence on interventions for APD in adults and establish the reliability of that data. While the data supporting PRMS is relatively robust, the evidence for AT and LGHA is less reliable due to the limitations mentioned. Therefore, the reliability of the data varies across interventions, highlighting the need for high-quality research to address these gaps.

We believe this review has the potential to provide researchers with the information required to plan high quality research that will answer many of the pressing questions regarding effectiveness of treatments for APD in the adult population.

## Data availability statement

The data analyzed in this study is subject to the following licenses/restrictions: Published data from previous research was used for this systematic analysis and is available upon request. Requests to access these datasets should be directed to NK, n.koohi@ucl.ac.uk.

## Author contributions

RC: Data curation, Formal analysis, Investigation, Methodology, Visualization, Writing – original draft, Writing – review & editing. SC: Data curation, Formal analysis, Investigation, Writing – review & editing. DK: Investigation, Writing – review & editing, Methodology, Validation, Visualization. PG: Investigation, Methodology, Validation, Writing – review & editing. D-EB: Investigation, Methodology, Validation, Writing – review & editing, Conceptualization, Data curation, Visualization, Writing – original draft. NK: Conceptualization, Data curation, Investigation, Methodology, Validation, Visualization, Writing – original draft, Writing – review & editing, Formal analysis, Project administration, Supervision.
