# Corrigendum: Hearing rehabilitation of adults with auditory processing disorder: a systematic review and meta-analysis of current evidence-based interventions

**DOI:** 10.3389/fnhum.2024.1468962

**Published:** 2024-08-13

**Authors:** Rachel Crum, Sanathorn Chowsilpa, Diego Kaski, Paola Giunti, Doris-Eva Bamiou, Nehzat Koohi

**Affiliations:** ^1^The Ear Institute, University College London, London, United Kingdom; ^2^Otology Neurotology and Communication Disorder Unit, Department of Otolaryngology, Faculty of Medicine, Chiang Mai University, Chiang Mai, Thailand; ^3^Department of Clinical and Movement Neurosciences, Institute of Neurology, University College London, London, United Kingdom; ^4^Neuro-otology Department, University College London Hospitals, London, United Kingdom; ^5^Ataxia Centre, National Hospital for Neurology and Neurosurgery, University College London Hospitals, London, United Kingdom; ^6^Biomedical Research Centre, National Institute for Health Research, London, United Kingdom

**Keywords:** auditory processing disorder, auditory training, low-gain hearing aids, personal remote microphone system, speech in noise perception

In the published article, there was an error in Figure 1, where an additional arrow was added to the PRISMA diagram. The corrected Figure 1 and its caption appear below.

**Figure 1:**
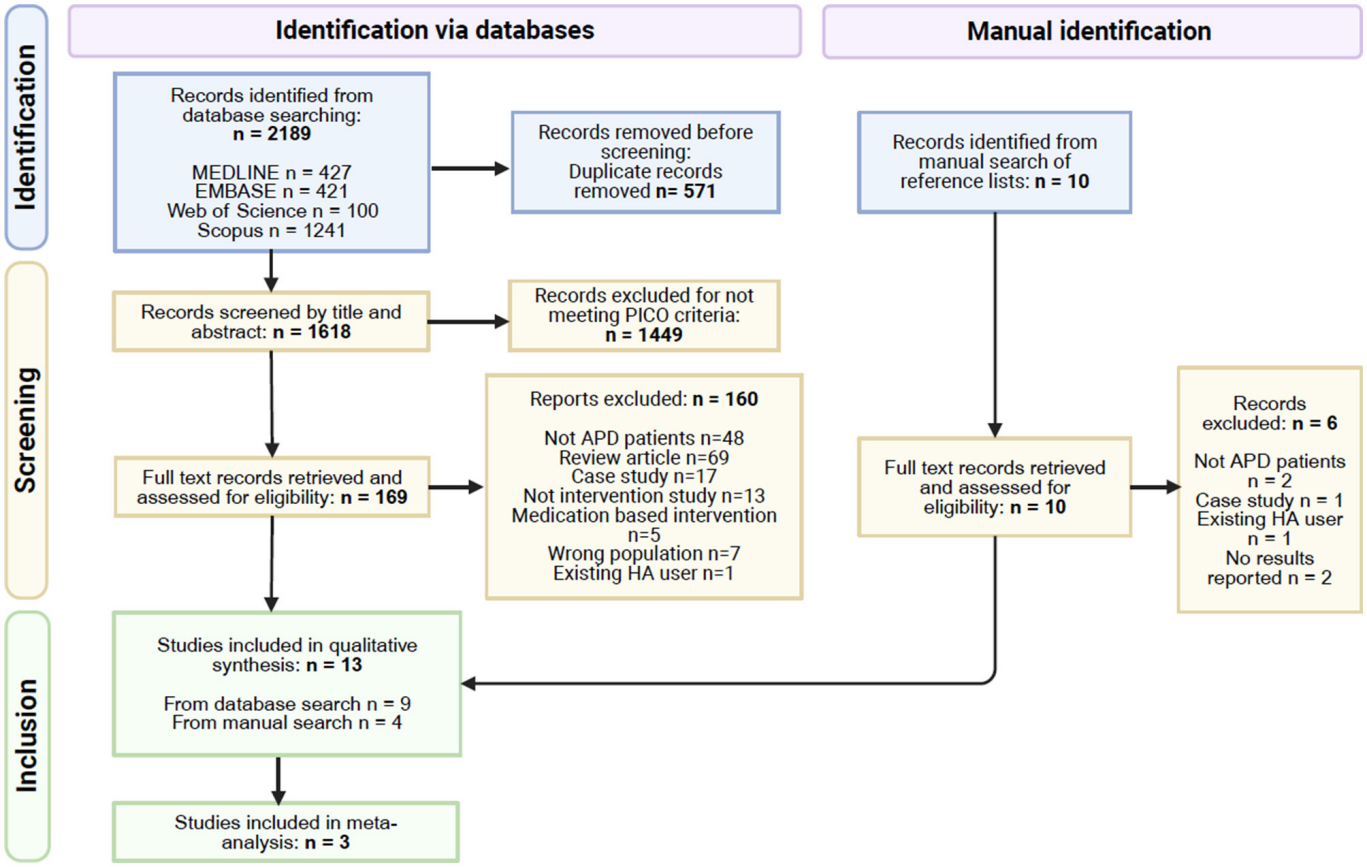
PRISMA flow diagram (Page et al., 2021), some studies had multiple reasons for exclusion.

The authors apologize for this error and state that this does not change the scientific conclusions of the article in any way. The original article has been updated.

