# Supplementary material for: Hearing rehabilitation of adults with auditory processing disorder: a systematic review and meta-analysis of current evidence-based interventions
Source: Front Hum Neurosci. 2024 Jun 21;18:1406916. doi: 10.3389/fnhum.2024.1406916 (PMC11224551; doi:10.3389/fnhum.2024.1406916)
Supplement: Supplementary file 1 [file Table_1.docx]

Supplementary Material

Hearing Rehabilitation of Adults with Auditory Processing Disorder (APD): A Systematic Review and Meta-analysis of Current Evidence-Based Interventions

Rachel Crum, Sanathorn Chowsilpa, Diego Kaski, Paola Giunti, Doris-Eva Bamiou, Nehzat Koohi^*^

*** Correspondence:** Nehzat Koohi: n.koohi@ucl.ac.uk

## Appendix A - GRADE evidence profile: use of personal remote microphones as an intervention for APD

| **Quality Assessment- Certainty of Evidence** | | | | | | | | |
| --- | --- | --- | --- | --- | --- | --- | --- | --- |
| **Outcome** | **No. of studies (design)** | **Limitations** | **Inconsistency** | **Indirectness** | **Imprecision** | **Publication bias** | **Certainty of the evidence (GRADE)** | **Comments** |
| SIN | 3 (3 non- randomised) | Serious limitations | Serious inconsistency | Serious indirectness | Serious imprecision | None detected | Low | Non-randomised, limitations.  Heterogeneity was substantial I^2^=83%. Populations had different co-morbidity. Lower end of confidence interval is 0.02 (very small effect) and all 3 studies had small sample sizes. Upgraded from very low as the studies were not case reports and did not have missing data. 2 of studies had serious risk of bias. |
